# Emerging Role of Antibody-Drug Conjugates and Bispecific Antibodies for the Treatment of Multiple Myeloma

**DOI:** 10.3390/antib11020022

**Published:** 2022-03-24

**Authors:** Waqqas Tai, Ahsan Wahab, Diana Franco, Zunairah Shah, Aqsa Ashraf, Qurrat-Ul-Ain Abid, Yaqub Nadeem Mohammed, Darshan Lal, Faiz Anwer

**Affiliations:** 1 Department of Internal Medicine, The Brooklyn Hospital Center, Brooklyn, NY 11201, USA; waqqastaido@gmail.com; 2 Department of Internal Medicine, Prattville Baptist Hospital, Prattville, AL 36066, USA; drahsan.wahab@gmail.com; 3 Department of Internal Medicine, Loyola MacNeal Hospital, Berwyn, IL 60402, USA; diana.Franco@luhs.org; 4 Department of Internal Medicine, Weiss Memorial Hospital, Chicago, IL 60640, USA; zshah@weisshospital.com; 5 Department of Internal Medicine, Northwell Health, Mather Hospital, Port Jefferson, NY 11777, USA; aqsaashraf18@gmail.com; 6 Department of Internal Medicine, AMITA Health Saint Joseph Hospital, Chicago, IL 60657, USA; qabidmd@gmail.com; 7 Department of Internal Medicine, St. Joseph Mercy Oakland Hospital, Pontiac, MI 48341, USA; drnadeemmd@gmail.com; 8 Department of Internal Medicine, University of Nevada School of Medicine, Las Vegas, NV 89102, USA; darshanlal86@hotmail.com; 9 Taussig Cancer Center, Myeloma Program, Cleveland Clinic, Cleveland, OH 44106, USA

**Keywords:** multiple myeloma, immunotherapy, antibody, targeted therapy, antibody drug conjugate, bispecific antibody

## Abstract

Multiple myeloma (MM) is characterized by malignant proliferation of malignant plasma cells; it is the second most common hematological malignancy associated with significant morbidity. Genetic intricacy, instability, and diverse clinical presentations remain a barrier to cure. The treatment of MM is modernized with the introduction of newer therapeutics agents, i.e., target-specific monoclonal antibodies. The currently available literature lacks the benefits of newer targeted therapy being developed with an aim to reduce side effects and increase effectiveness, compared to conventional chemotherapy regimens. This article aims to review literature about the current available monoclonal antibodies, antibody-drug conjugates, and bispecific antibodies for the treatment of MM.

## 1. Introduction

Multiple myeloma (MM) is a malignant proliferation of plasma cells (PCs) that produces excess monoclonal immunoglobulins [1]. Myeloma cells prevent normal antibody production, leading to the accumulation of abnormal monoclonal immunoglobulins that compromise the body’s immune response, making it susceptible to infections. MM is the second most common hematological malignancy, accounting for 13% of blood cancers [2]. PC dyscrasias include a wide range of diseases including an asymptomatic premalignant proliferation of PCs such as monoclonal gammopathy of undermined significance (MGUS), asymptomatic smoldering multiple myeloma (SMM), and symptomatic malignant diseases such as MM and plasma cell leukemia with extensive end-organ damage [3,4]. Diagnosis and follow-up can be challenging; not all MGUS develop into MM, and some do transform to Waldenstrom’s macroglobulinemia, primary AL amyloidosis, POEMS syndrome, or a lymphoproliferative disorder [5]. The rate of progression of PC disorder from MGUs to MM or a related disorder is approximately 1% each year [6]. MM treatment is based on end-organ damage and markers of active disease [7]. As per the Surveillance, Epidemiology, and End Results (SEER) data, with advancements in treatment options, the survival rates have improved from 25% in 1975 to over 50% by 2011 and with further therapeutic options, ten-year survival is possible [8]. The responses to treatment options and the survival benefits of newly diagnosed multiple myeloma (NDMM) are heterogeneous, ranging from 2 to >10 years [9]. Remarkable advancements in the understanding of the pathophysiology have revolutionized treatment options and patient outcomes. Preclinical studies focusing on pathophysiology of MM have prompted the development of more effective targeted therapies. However, genetic intricacy, instability, and diverse clinical presentations of MM remain ongoing barriers to cure. The treatment of MM has been modernized with the introduction of newer therapeutic agents, i.e., target-specific monoclonal antibodies. This article aims to review the current available monoclonal antibodies (mAbs), Antibody-Drug Conjugates (ADCs), and bispecific antibodies for the treatment of MM.

## 2. Role of Monoclonal Antibodies in RRMM

The underlying pathophysiology of MM formation, which involves immune dysregulation, has led to investigational treatment strategies using mAbs targeting the myeloma cells. The current standard for MM management involves mAb use in the majority of three-drug regimens used for initial and subsequent therapy. mAbs are routinely used for both NDMM and relapsed/refractory multiple myeloma (RRMM). RRMM definition is based on the recommendation of International Myeloma Working Group (IMWG), as the disease recurs after the initial response and manifests as the development of new plasmacytomas or hypercalcemia, ≥25% increase of the urine or serum monoclonal protein (M-protein), or the development of new hypercalcemia or plasmacytomas [10]. Relapse is defined as an increase in bone marrow PCs in patients with the non-secretory disease. MM that becomes progressive or non-responsive to therapy within 60 days of the last treatment and has achieved a minimal response is defined as RRMM [11]. The safety and toxicity data while using mAbs for MM treatment are given below (Table 1).

### 2.1. Daratumumab (Anti-CD38)

Daratumumab (anti-CD38) was the first mAb approved for MM treatment and has been most extensively studied. The GEN501 and SIRIUS studies have investigated its use as a single agent for RRMM [12,13]. Pooled post hoc analysis of both the trials reported overall response rate (ORR) of 31.1%, progression-free survival (PFS) 4 months, with an approximate overall survival (OS) of 20 months [14]. The frequency of grade ≥ 3 toxicity reported in these studies was 2 to 8.9% with hypertension, pneumonia, and anemia found to be the most common. Pomalidomide which upregulates CD38 expression was studied in combination with daratumumab by Chari et al. in RRMM and demonstrated improved ORR of 60%, PFS of 8.8 months, with an OS of 17.5 months [15]. Approximately 2 to 8% reported grade ≥ 3 toxicity, with dyspnea being the most common. The combination of daratumumab with other agents led to promising results.

GRIFFIN, a phase II trial in NDMM, while evaluating daratumumab in combination with bortezomib, lenalidomide, and dexamethasone (Dara-VRd) demonstrated an ORR of 99% vs. 91.8% and 24-month PFS of 95.8% vs. 89.8%, in daratumumab and the control arm, respectively [16]. Although the frequency of grade ≥ 3 was higher in the daratumumab group (thrombocytopenia 16.2% vs. 8.8%, neutropenia 41.4% vs. 21.6%, and infections 90.9% vs. 61.8%), however, the rate of treatment discontinuation was lower in the daratumumab group (15.2% vs. 20.6%). Noteworthy phase III trials for NDMM include ALCYONE, MAIA, and CASSIOPEIA [17,18,19]. All of these showed ORR of greater than 90% in the daratumumab group whereas it ranged from 73.9 to 89.9% in the control arm. In the ALCYONE trial, the 36-month OS was 78.0% vs. 67.9%, respectively. The most common adverse events (AEs) included neutropenia (15–50%) and infections (14.7–32.1%) and these were encountered more frequently in the daratumumab group in all studies. However, except for infections, AEs were comparable in both groups.

Similarly, three studies (phase III trials) evaluated the mAb combinations for RRMM [20,21,22]. CASTOR trial reported an ORR of 83.8% vs. 63.2% while the POLLUX study reported 92.9% vs. 76.4% in the daratumumab vs. control group, respectively. Similarly, PFS (in months) in% in daratumumab vs. control group was 16.7 vs. 7.1 and 44.5 vs. 17.5 in CASTOR and POLLUX studies, respectively. The CANDOR trial reported an ORR of 84% in the daratumumab group and 75% in the control group. Daratumumab is usually administered via intravenous (IV) route and approximately half of the patients experience infusion-related reactions (IRRs). Therefore, PAVO (a phase Ib trial), showed promising findings when daratumumab was administered subcutaneously (SC) [23]. A phase III study, COLUMBA, later confirmed better ORR of 41% vs. 37% and fewer IRRs (13% vs. 34%) in SC vs. IV route, respectively [24]. The PLEIADES study showed that SC daratumumab retained its efficacy when used in combination with other agents [25].

### 2.2. Isatuximab

Isatuximab is another mAb targeting CD38. Preliminary trials (phase I and II) showed an ORR of 56 to 62.6% for RRMM patients [26,27,28]. GMMC-CONCEPT trial on NDMM patients showed an ORR of 100%. The ICARIA-MM and IKEMA (phase III trials) showed ORRs of 60% vs. 35% and 86.6% vs. 82.9% in isatuximab-combination vs. control group, respectively, in RRMM patients [29,30]. In the ICARIA-MM study, PFS (months) was 11.5 for isatuximab vs. 6.5 in control group. Commonly reported AEs of grade 3 from these studies included neutropenia (7.4–85%), respiratory infections (14–32.2%), thrombocytopenia (23.8–29.9%), and cardiac failure (4%). Newer anti-CD38 mAbs including MOR202 and TAK-079 are under investigation for their anti-MM efficacy.

### 2.3. Elotuzumab (Anti-SLAMF7)

Elotuzumab (anti-SLAMF7) in the ELOQUENT-2 and ELOQUENT-3 trials showed anti-MM activity in combination with other agents in RRMM patients [31,32]. ORR and PFS were 79% vs. 66% and 19.4 vs. 14.9 in elotuzumab group vs. control group, respectively, in ELOQUENT-2 study while these were 53% vs. 26% and 10.3 vs. 4.7 in ELOQUENT-3 trial, respectively. Neutropenia, infections, and hyperglycemia were the most common AEs reported. Elotuzumab has the lowest IRRs and is a safe option for frail patients with MM.

### 2.4. Pembrolizumab (Anti-PD-1)

Pembrolizumab (anti-PD-1) when used in combination with other agents showed initial promising results; however, safety concerns led to the cessation of its development for MM. The KEYNOTE-023 and HP-00061522 trials enrolling RRMM patients showed an ORR of 44% and 60%, respectively, while PFS was 7.2 and 17.4, respectively [33,34]. Neutropenia, hyperglycemia, and pneumonia were the most reported AEs. There are many other mAbs that have not been approved by the FDA yet exhibit activity such as anti-CD-40, anti-CD-38, and anti-CD74. Studies are underway evaluating the efficacy for anti-MM role of such agents.

**Table 1:** Monoclonal antibody summary of efficacy and grade 3 adverse events for the treatment of multiple myeloma.

Monoclonal Antibody	Study Name/Phase	Single Agent vs. Combination	Efficacy			Most Common Grade ≥ 3 Toxicity (%)
			ORR (%)	PFS (Months, %)	OS (Months, %)	
Daratumumab	GEN501 + SIRIUS Phase II [14]	Single agent	31.4%	19.6% (3-year PFS)	20.5 m, 36.5% (3-year OS)	Anemia (18%), thrombocytopenia/neutropenia (14%), hypertension (5%), back pain/hypercalcemia (3%), fatigue (3%)
	Chari et al. Phase I [15]	Combination with pomalidomide/dexamethasone	60%	8.8 m	17.5 m, 66% (1-year OS)	Neutropenia (78%), anemia (28%), and leukopenia (24%)
	GRIFFIN Phase II [16]	Combination with bortezomib/lenalidomide/dexamethasone	99%	-	-	Neutropenia (41.4%), peripheral neuropathy/diarrhea (7.1% each)
	ALCYONE Phase III [17]	Combination with bortezomib/melphalan/prednisone	90.9%	50.7% (3-year PFS)	78% (3-year OS)	Neutropenia (39.9%), infections (23.1%), IRRs (4.9%)
	MAIA Phase III [18]	Combination with lenalidomide/dexamethasone	92.9%	-	-	Neutropenia (50%), infections (32.1%), fatigue (8%)
	CASSIOPEIA Phase III [19]	Combination with bortezomib/thalidomide/dexamethasone	92.6%	-	-	Neutropenia (28%), stomatitis (13%), peripheral neuropathy (9%)
	CASTOR Phase III [20]	Combination with bortezomib/dexamethasone	83.8%	60.7% (1-year PFS)	-	Thrombocytopenia (45.7%), pneumonia (9.9%), hypertension (6.6%)
	POLLUX Phase III [21]	Combination with lenalidomide/dexamethasone	92.9%	85.7% (1-year PFS)	92.1% (1-year OS)	Neutropenia (55.5%), pneumonia (15.2%), diarrhea (9.9%)
	CANDOR Phase III [22]	Combination with carfilzomib/dexamethasone	84%	-	-	Thrombocytopenia (24%), respiratory infection (29%), hypertension (18%)
	PAVO Phase Ib [23]	Single agent (SC route)	42.2%	-	-	Anemia (15.6%), hypertension (8.9%), pneumonia/RSV/hyponatremia (4.4% each), device-related infections (4.4%)
	COLUMBA Phase III [24]	Single age (SC vs. IV)	41% for SC vs. 37% for IV	5.6 m for SC vs. 6.1 m for IV.	-	Anemia (13% SC vs. 14% IV), neutropenia (13% SC vs. 8% IV), thrombocytopenia (13% in SC vs. IV), pneumonia (3% SC vs. 4% IV)
	PLEIADES Phase II [25]	SC in combination with bortezomib/lenalidomide/dexamethasone	89.6%			Neutropenia (37.3%), lymphopenia (22.4%), thrombocytopenia (43.3%), injection-site reactions (7.5%)
Isatuximab	TCD11863 Phase I [26]	Combination with lenalidomide/dexamethasone	56%	8.5 m		Neutropenia (60%), pneumonia (9%), fatigue (7%)
	TCD14079 Phase I [27]	Combination with pomalidomide/dexamethasone	62.2%	17.6 m	-	Neutropenia (84%), pneumonia (18%), fatigue/urinary tract infection/traumatic fracture (7% each), syncope/dyspnea/hypertension (7% each)
	GMMC-CONCEPT Phase Ib [28]	Combination with carfilzomib/lenalidomide/dexamethasone	100%	-	-	Neutropenia (34%), hypertension (12%), cardiac failure (4%)
	ICARIA-MM Phase III [29]	Combination with pomalidomide/dexamethasone	60%	11.5 m	-	Neutropenia (85%), pneumonia (16%), dyspnea (4% vs. 1%)
	IKEMA Phase III [30]	Combination with carfilzomib/dexamethasone	86.6%	-	-	Respiratory infections (32.2%), cardiac failure (4%), thrombocytopenia (29.9%), neutropenia (19.2%)
Elotuzumab	ELOQUENT-3 Phase III [31]	Combination with pomalidomide/dexamethasone	53%	10.3 m	-	Neutropenia/infections (13% each), hyperglycemia (8%)
	ELOQUENT-2 Phase III [32]	Combination with lenalidomide/dexamethasone	79%	19.4 m	30%	Lymphopenia (79%), infections (33%), pneumonia (14%)
Pembrolizumab	KEYNOTE-023 Phase I [33]	Combination with lenalidomide/dexamethasone	44%	7.2 m	-	Neutropenia (27.4%), hyperglycemia/pneumonia (6.5% each), atrial fibrillation/insomnia (3.2% each)
	HP-00061522 Phase I/II [34]	Combination with pomalidomide/dexamethasone	60%	17.4 m	-	Neutropenia (42%), hyperglycemia (21%), fatigue (15%), pneumonia (15%)

Abbreviations: m: months; IRRs: infusion-related reactions; IV: intravenous; PFS: progression-free survival; RSV: respiratory syncytial virus; ORR: overall response rate; OS: overall survival; SC: subcutaneous.

## 3. Antibody-Drug Conjugates (ADC)

### 3.1. Belantamab Mafodotin

B-cell maturation antigen (BCMA) is a cell surface protein expressed on PCs that has shown promise as a potential candidate for targeted therapy of RRMM. BCMA has the potential to maintain long-lived PC levels. Multiple studies have shown that BCMA^−/−^ mice are unable to produce naïve B cells and PCs; however, memory B-cell levels remain intact [35,36,37]. BCMA levels are elevated in MM and may play a role in prognostication [38,39]. Additionally, BCMA levels progressively increase from MGUS to SMM to active MM [40]. Knowing that knocking out BCMA can inhibit the progression of PCs, we hypothesized that disturbing BCMA may function as a therapeutic modality in MM. Tai et al. developed a novel anti-BCMA mAb which was identified as J60M. They evaluated J60M with a drug conjugate, monomethyl auristatin E, an anti-microtubule drug that showed multiple mechanisms of action against MM cells [41]. This ADC is named belantamab mafodotin (anti-BCMA with a monomethyl auristatin F payload). This study subsequently led to the DREAMM clinical trials, which were a multiphase clinical trial program to evaluate drug efficacy, safety, dosing, and comparison to the standard of care in RRMM. This review will focus primarily on efficacy and toxicity of ADCs including belantamab mafodotin (Table 2).

The DREAMM-1 clinical trial focused on patient satisfaction through to the end of trial interview and the initial safety profile. Patients reported a mean satisfaction score of 7.9/10 using bone pain as their marker. The most common side effect was blurred vision from keratopathy with 8/13 patients reporting resolution or improvement of symptoms after end of trial [42]. The DREAMM-2 trial was a multicenter study that focused on AEs and outcomes in intention-to-treat populations [43]. This study used the IMWG consensus criteria to grade outcomes [44]. They concluded that among patients who received belantamab mafodotin, 19% had a very good partial response (VGPR) in the low-dose group (2.5 mg/kg), and a 20% “stringent complete (sCR) or complete response (CR)” in the large-dose group (3.4 mg/kg). In terms of toxicity, most common AE in grades 3–4 was keratopathy in 27% of patients in the lower-dose group, and 21% in the high-dose group. Additionally, in low vs. high dose, thrombocytopenia occurred at 20% and 33%, anemia occurred at 20% and 25%, respectively. The DREAMM-9 study focused on belantamab mafodotin and compared it to the bortezomib, lenalidomide, and dexamethasone (VRd) regimen which is the standard of care for transplant-eligible and transplant-ineligible NDMM. They compared bortezomib or LBJ in combination with belantamab mafodotin. AEs occurred in all 12 patients in this study, including thrombocytopenia, neutropenia, and corneal events. Grade ≥ 3 AEs happened in 75% of the patients; all of them required dose reduction and delay. All patients achieved a VGPR response at minimum with 25% achieving sCR and 42% achieving CR. The DREAMM-9 study is still being continued but preliminary data suggest that there are no new AEs and full efficacy will be evaluated at the end of the entire study [45]. The DREAMM trials 4–10 are currently in progress and will focus on comparing the standard of care regimens to belantamab and to other therapies to develop multidrug therapeutic regimens [46].

A recent case series was published characterizing the response to belantamab mafodotin and the corneal events associated with treatment [47]. Five patients were evaluated in this study. Corneal events were common among several participants, including dry eyes, photophobia, and keratopathy. All patients were prescribed ocular lubricants and steroid eye drops. This is contrary to the DREAMM-2 study which reported no benefit from administering prophylactic steroid eye drops for keratopathy; however, patients reported relief of ocular symptoms. Ocular complaints seemed to be dose-dependent, and as such, dose modification including delay and reduction played a role in improving corneal events.

Belantamab mafodotin is a novel therapy that has been approved by the FDA for RRMM. Several clinical trials are still in place to determine the role of belantamab mafodotin in current chemotherapy regimens. Further reviews will need to be conducted in the next one to two years pending the completion of the numerous trials concurrently running.

### 3.2. Lorvotuzumab Mertansine

Lorvotuzumab mertansine (IMGN901; LM) is an anti–CD56-DM ADC that is composed of humanized ant-CD56 antibody lorvotuzumab (huN901) which is linked to the cytotoxic and anti-mitotic agent drug maytansinoid 1 (DM1) [48]. CD56 is a cell surface glycoprotein originally identified as a neural cell adhesion molecule (NCAM, which plays a role in cell adhesion and mediates cell-cell and cell-matrix interactions [49]. It is generally not present in healthy PCs; however, its overexpression in malignant PCs has been identified in >78% of MM patients [50,51]. It has also been shown that CD56-negative MM is associated with reduced bone destruction, increased frequency of extramedullary spread, and plasma cell leukemia [52].

In vitro and in vivo testing of anti-CD56 antibodies such as humanized mAb huN901 showed potent anti-myeloma activity. In this preclinical study in mouse models, LM impaired the survival of CD56-expressing MM cells in a dose-dependent manner by causing cytotoxicity associated with G2-M cell cycle arrest and leading to apoptosis, while CD56-negative tumor cells remained unharmed. These results support the clinical trials for the antibody-drug conjugate LM, which targets CD56-positive MM cells [53]. Using the CD56 target, Benjamin et al. designed the anti-CD56 CAR-T-cells to target CD56 MM cells in another preclinical study. These results demonstrated for the first time the impressive anti-tumor effect of anti-CD56 targeted antigen receptors in a systemic xenograft model of myeloma [54]. Phase I monotherapy trials by Chanan-Khan et al. for CD56-positive RRMM showed an ORR of 7%. The toxicity profile was predictable as reported in other studies and consisted mostly of peripheral neuropathy, fatigue, and cytopenia [55].

These encouraging preclinical results led to a phase I trial. In a study by Ailawadi et al., LM was evaluated as a single agent in 37 RRMM CD56-positive patients (after failing more than three prior treatments in 78% of patients). The maximum tolerated dose (MTD) was 112 mg/m^2^ after two patients experienced dose-limiting toxicity (DLT) at 140 mg/m^2^, symptoms including grade 3 fatigue and grade 3 renal failure. In total, 51% of patients experienced treatment-related peripheral neuropathy (5.3% grade 3–4). The AE profiles were manageable, with low incidence of AEs and no IRRs [56]. In a second phase 1 trial by Berdeja et al., 44 patients with RRMM patients (after failing two prior lines of treatment; 33% lenalidomide refractory), were administered the combination of LM with lenalidomide and dexamethasone in a four-week cycle. The combination therapy resulted in ORR of 59% with an sCR including eight patients with a VGPR or better and nine patients with “partial remission”. The side effects profile again included neuropathy (mostly grade 2 or less) as the most common AE. The ORR of 59% was considered insufficient and dose-related peripheral neuropathy was considered to be a significant AE, which led to the discontinuation of further studies on this combination [57].

In a phase I/II study, LM alone or in combination showed promising results against CD56-positive MM which revealed additive to synergistic anti-MM effects using combination therapy with lenalidomide, dexamethasone and bortezomib [58]. Lastly, CD56 CAR-T-cell therapy is being investigated, in combination with other antigens that are present on MM cells. A clinical trial by Yanjie Hi on CAR-T-cell therapy in RRMM was terminated due to difficulties in enrolment and questionable benefits (NCT03473496). Another clinical trial (NCT03271632) is in the recruitment process.

### 3.3. HDP-101

HDP-101 is a newer class of anti-BCMA ADC named as antibody-targeted amanitin conjugate (ATAC) that is fused to the RNA polymerase inhibitor amanitin via a non-cleavable linker. BCMA is selectively present on the MM cells and hence is considered an ideal target for amanitin-based ADCs [59]. Using a novel anti-BCMA ADC (HP-101), Figueroa et al. determined the level of antibody-bound molecules against BCMA using anti-BCMA (HDP-10). They demonstrated that upon binding to BCMA, ≥60% of bound HDP-101 was internalized within 2 h in MM cell lines [60]. Based on the highly favorable results from their study, HDP-101 was recently (May 2021) advanced to a phase I safety assessment trial (NCT04879043) in RRMM patients. Amanitin is an extremely potent RNA polymerase II inhibitor that impedes the cellular transcription even at very low concentrations regardless of the proliferating or resting status of the cells, resulting in cell death [61]. This means that it is toxic even to resting MM cells; it is an important property given that a large fraction of MM cells do not proliferate in many cases. This rare property is a clinically important characteristic of HDP-101 and makes it superior to the other microtubule inhibitors which mainly kill only proliferating cells [62]. Amanitin is chemically produced from the deadly *Amanita phalloides* (“green death cap”) mushrooms [63].

Preclinical studies have demonstrated that even pico- or nanomolar concentrations of HDP-101 are extremely cytotoxic to BCMA-positive myeloma cells and non-proliferating primary MM cells isolated from RRMM irrespective of BCMA level of expression. HDP-101 treatment also caused significant dose-dependent tumor regression including complete remission in mouse xenograft models with both subcutaneous and systemic MM [64].

In vivo studies in non-human primates have demonstrated favorable safety profiles including mild to moderate increases in liver enzymes and LDH [65,66]. Deletion (del) of 17p involving the p53 tumor suppressor (*TP53*) gene is an adverse prognostic factor in MM. A preclinical study performed by Singh et al. demonstrated efficacy of HDP-101 in cell lines with a deletion of 17p.

The success of multiple preclinical and non-human studies led to the initiation of in-human studies. Strassz et al. initiated the first-in-human study of HDP-101 which is currently in progress [67]. Another clinical trial to assess the safety of HDP-101 in patients with RRMM that started in May 2021 is under clinical evaluation in a phase I/II trial in patients with RRMM (NCT04879043).

### 3.4. Anti-ICAM1

Intercellular cell adhesion molecule 1 (ICAM1) is highly expressed in the vast majority of MM cells compared to that in normal cells. with the exception of progenitor cells, ICAM1 expression is absent in most normal hematopoietic cells. PCs from patients already treated with chemotherapy and with multidrug resistance phenotype also express higher levels of ICAM-1 [68]. Naked anti-ICAM1 antibodies were active in preclinical studies and safe in myeloma patients but showed limited clinical efficacy; thus, an anti-ICAM1 ADC was developed for better targeting MM cells. Potent anti-myeloma cytotoxicity was displayed by anti-ICAM1 ADC in vitro and in vivo [69,70]. In a study conducted by Hansson et al. (NCT010252060), BI-505, a human anti-ICAM-1 mAb, was evaluated for safety and tolerability in advanced RRMM patients. Thirty-five patients with six prior lines of therapy were enrolled and no objective response was observed. AEs were observed in 97%. Grade 3 AEs were reported in 8% of patients. The most common treatment-related AEs were reversible IRRs, pyrexia, chills, and headache [71]. In a phase II study by Wichert et al. (NCT01838369), efficacy of BI-505 was evaluated in patients with SMM, four patients were enrolled, and three patients completed the first cycle, and no objective response was reached [72]. In a study reported by Klausz et al., anti-ICAM-1 antibody MSH-TP 15 developed by engineering a fragmented crystallizable (Fc) part of the antibody improved the recruitment of immune effector cells [73].

### 3.5. AMG 224

AMG 224 is a relatively naïve drug that is being investigated. AMG 224 consists of an anti-human BCMA IgG1 antibody conjugated with mertansine (DM1), an anti-tubulin maytansinoid, through a non-cleavable linker. Hans et al. conducted the first phase I trial to investigate the safety and efficacy of AMG 224 in patients with RRMM [74]. The final outcome of the study was an ORR of 23%. In this study, 5% sCR, 5% had a VGPR, 13% had a partial response (PR), and lastly, 15% had progression of the disease. In terms of AEs, 31% developed grade 3 thrombocytopenia. The development of ocular complications is the primary outcome of ADCs. In this study, no ocular events, however, were noted. The authors noted that in comparison to belantamab mafodotin, AMG 224 had lower incidence of ocular events, but higher incidence of thrombocytopenia [75]. This study provides evidence to support the safety of AMG 224 and its ability to result in a significant therapeutic response.

### 3.6. ABBV-838

ABBV-838 is an ADC comprising a humanized recombinant anti-CS1 immunoglobulin G1κ conjugated to the cytotoxic monomethyl auristatin E (MMAE) [76]. In vivo mouse models have shown that CS1 is a cell-surface glycoprotein that is expressed on myeloma cells in >90% of cases. Anti-CS1 antibody staining of the bone marrow showed myeloma and natural killer (NK) cells as the primary targets [77]. Furthermore, in vitro and in vivo studies support this claim, showing that anti-CS1 antibodies inhibit MM cell binding to stromal cells in the bone marrow and induce cellular cytotoxicity against the myeloma cells [78,79]. This provides a proof of the concept that anti-CS1 antibodies have therapeutic potential. Ravi et al. conducted a human phase I clinical trial of ABBV-838 [47]. The most common AE reported was that 28% of the participants developed neutropenia and anemia. Of note, 16% of patients developed corneal deposits, and 17.3% developed thrombocytopenia. In terms of efficacy, 8% achieved VGPR, 2.7% achieved PR, 69.3% had the best overall response, and 18.7% of patients had progressive disease.

### 3.7. Anti-FcRH5

Fc receptor homolog 5 (FcRH5) is a cell surface antigen of unknown function that is expressed only in mature B cells. MM cells express higher levels of FcRH5 than normal B cells. Therefore, it is a potential site for targeted therapeutics. DFRF4539A is an anti-FcRH5 mAb conjugated to monomethyl auristatin via a cleavable linker, a potent antimitotic agent [80,81,82]. The drug showed limited activity in the phase I trial by Stewart et al. and high incidence of toxicity. In this phase I trial (NCT01432353), DFRF4539A was administered to patients as a single agent at 0.3–2.4 mg/kg every 3 weeks or 0.8–1.1 mg/kg weekly. About 95% of patients developed AEs; the most common AE was anemia, 26%, followed by fatigue, 21%. Approximately 12% developed grade 3 AEs; the most common grade 3 AE was neutropenia in 10% of patients; no deaths were reported [83]. Further investigation of DFRF4539A was stopped owing to the limited activity observed in this study; currently, no clinical trial is ongoing using DFRF4539A. FcRH5 may be a potential myeloma target in the future in drugs that have other mechanisms of action when directed against FcRH5 and hence may prove beneficial to patients with MM.

**Table 2:** Antibody drug conjugates summary of efficacy and grade 3 adverse events for treatment of multiple myeloma.

ADCs	Study Name/Phase	Single Agent vs. Combination	Efficacy		Most Common Grade ≥ 3 Toxicity (%)
			ORR (%)	PFS (Months, Weeks)	
Belantamab mafodotin	DREAMM-2 Phase II [43]	Single agent (low vs. high dose)	31% vs. 34%	2.9 m vs. 4.9 m	Keratopathy (27% vs. 21%), thrombocytopenia (20% vs. 33%), anemia (20% vs. 25%)
	DREAMM-9 Phase I [45]	Single agent vs. standard of care	-	-	Thrombocytopenia/neutropenia (100% each), keratopathy (100%)
Lorvotuzumab mertansine	Chanan-Khan et al. Phase I [55]	Single agent	17.9%	-	Fatigue/weakness/peripheral neuropathy/renal failure (1 each)
	Ailawadi et al. Phase I [56]	Single agent	-	26.1 weeks	Fatigue (5.4%), areflexia/peripheral neuropathy/neutropenia (2.7%)
	Berdeja et al. Phase I [57]	Lorvotuzumab + lenalidomide/dexamethasone	59%	-	Tumor lysis syndrome (10%), neutropenia/thrombocytopenia/anemia (5% each), hemolytic anemia/LDH increase (5% each)
HDP-101	Strassz et al. Phase I/IIa [67]	Single agent (human)	-	-	-
Anti-ICAM1	Hansson et al. Phase 1 [69]	Single agent (human)	-	-	-
AMG 224	Hans et al. Phase I [74]	Single agent	23%	-	Thrombocytopenia (31%)
ABBV 838	Ravi et al. Phase I [76]	Single agent	10.7%	-	Neutropenia/anemia (28% each), thrombocytopenia (17.3%), keratopathy (16%)
Anti-FcRH5	Stewart et al. Phase I [81]	Single agent	-	-	Neutropenia/infections (10.3% each), nervous system disorder (7.7%)

Abbreviations: ADCs: antibody-drug conjugates; PFS: progression-free survival; m: months; ORR: overall response rate; OS: overall survival.

## 4. Bispecific Antibodies (BiAbs)

### 4.1. Teclistamab

Teclistamab (also known as JNJ-64007957 or JNJ-7957) is another BCMA × CD3 bispecific T-cell engager IgG4 antibody that was developed using Genmab DuoBody technology and mediates T-cell-redirected killing of myeloma cells [84]. BCMA-expressing myeloma cells showed a robust but specific elimination in preclinical studies (xenograft models, myeloma cell models from heavily pretreated patients) when incubated with T cells and teclistamab, therefore encouraging first-in-human clinical trial of teclistamab (MajesTEC-1, NCT03145181) [85,86]. Although AMG-420 showed a potent clinical response in MM, it had a shorter half-life necessitating continuous infusion for 4 weeks precluding its use in clinical settings [87]. In contrast to AMG-420, teclistamab has a longer half-life with a sustainable blood level up to 10 days and therefore can be administered as once-weekly convenient dosing. MasjesTEC-1 was a single-arm phase I study completed in two phases in RRMM patients (median therapies: 6) to evaluate the recommended phase 2 dose (RP2D) and its safety and efficacy at RP2D. Teclistamab was given either IV (biweekly cohort: 0.3–19.2 µg/kg, once-weekly cohort: 19.2−720 µg/kg) or SC (once-weekly: 80−3000 µg/kg). One hundred and fifty-seven enrollees (82% triple-refractory, 39% penta-refractory, and 92% refractory to last therapy) in the trial had received at least a single dose of the drug either as IV (*n*: 12 biweekly + 72 once-weekly: 84) or SC (*n*: 73). Two dose-limiting toxicities occurred with IV doses (stage 4 delirium in one patient and stage 4 thrombocytopenia in the context of CRS and DIC in second patient). RP2D identified was SC once-weekly at 1500 mg/kg in 40 patients (median age 63, range: 57–69, refractory to PI: 88%) after step-up doses and had no dose-limiting toxicity. Most common treatment AEs were CRS in 70% (no grade ≥ 3) and neutropenia in 65% (grade ≥ 3: 40%) of patients at RP2D level. These percentages were 57% (no grade ≥ 3) and 61% (grade ≥ 3: 48%), respectively, for the entire cohort of 157 patients. The majority of these AEs occurred in step-up dosing or cycle 1. When CRS occurred at RP2D, 35% and 13% of patients received tocilizumab and steroids as treatment, respectively. Notably, teclistamab developed CRS after a median of one day whereas CRS occurred after a median of two days in the SC formulation. CRS overall rate was 57% irrespective of dose or route. Infusion-related reactions were 5% and occurred among those who received teclistamab intravenously. Injection-related reactions, however, were reported only in the SC route with occurrence in 42% of 73 patients. Those who received RP2D had an overall response of 65%, VGPR of 58%, and CR 40%. Treatment discontinuations occurred at other doses (6%) vs. none for RP2D.

A phase II study of MajesTEC-1 (NCT04557098) is ongoing with RP2D with the main goal of ORR estimation before 30 December 2024 [88]. Evaluation of blood samples from patients who showed a clinical response to teclistamab revealed lower soluble BCMA (sBCMA) levels post-treatment [89]. Similarly, those who relapsed on teclistamab showed a decline in soluble BCMA levels followed by an upward trend in sBCMA correlating with relapse. Preclinical data also show that patients (evaluated bone marrow samples) who were previously treated with daratumumab may have enhanced anti-myeloma activity with teclistamab compared to other patients without previous daratumumab exposure.

Two phase-1 dose-escalation studies (NCT04586426 and NCT04108195) are evaluating the various combinations of teclistamab and talquetamab with or without daratumumab and with or without pomalidomide. A phase III clinical trial (NCT05083169) is comparing teclistamab-daratumumab combination with daratumumab-pomalidomide-dexamethasone and daratumumab-bortezomib-dexamethasone [90].

### 4.2. CC-93269

CC-93269, previously known as EM801, is a trivalent (2:1) T-cell engager IgG antibody which is a bispecific binder for the BCMA antigen on myeloma cells (bivalent binding) and CD3 epsilon receptors on T cells (monovalent binding) [91]. The trivalent structure of CC-93269 with simultaneous attachment to both BCMA and CD3+ epsilon receptors causes T-cell myeloma cell interaction, activating CD3 downstream signaling pathways and increasing the expression of CD25 and CD69, thereby releasing granzyme B and proinflammatory cytokines such as IFN-γ or TNF-α [92]. These cytokines from redirected T cells attack myeloma cells and induce apoptosis. Following the successful demonstration of myeloma cell regression in animal and cell models, the first human trial (NCT03486067) started in 2018 aiming to determine the best dose for myeloma patients [93]. Interim results were presented in May 2020 with a cutoff date for data from 28 October 2019 [94]. Thirty patients who received a median of 3.5 cycles (range: 1–12) had heavily pretreated and refractory disease (median therapies: 5, range: 3–13, 88% refractory to immunomodulators as last therapy, 77% refractory to daratumumab and proteasome inhibitors as last therapy) with a median age of 64 years and median duration of 5.9 years following diagnosis. None of the patients had previously received BCMA therapy. Treatment-emergent AEs of grade 3–4 occurred in 73% of patients (*n*: 22) such as neutropenia (43%), anemia (37%), thrombocytopenia (17%), and infections (30%). To be noted, CRS was quite common in the trial, involving 77% (*n*: 23) of patients, and the majority of events occurred with the first or second dose of CC-93269 (95.7% of events) [95]. Among the 22 patients with 37 CRS events, 30 events had a maximum grade of 1 or 2. The study already had CRS prophylaxis protocol with dexamethasone before the first dose of CC-93269 and those with 6 mg or above. When CRS occurred, 73% of events were treated with dexamethasone and 43% with tocilizumab. One patient had died due to CRS and infection. That patient received first dose of 6 mg and second dose of 10 mg on the 8th day of the cycle. ORR was 43%, and CR/sCR was 17%. Among a subpopulation of patients (*n*: 9) who received 10 mg of the drug, ORR was 89% with 44% of patients having sCR/CR. Therefore, the response was dose-dependent and improved at higher doses. About 11 responses remained stable during follow-up for 5.3 to 40.6 weeks. Among 13 patients with ORR, MRD-negativity was achieved in 92% of patients. The trial is ongoing with the aim of determining the dose for phase II.

### 4.3. AMG 420

AMG 420 (Amgen), a bispecific T-cell engager (BiTE), formerly known as BI 836909, targets BCMA on MM cells and causes T-cell-mediated lysis [96,97]. Topp et al. tested AMG 420 in 42 MM patients who had received a median of four prior therapies. They concluded that this drug could be an emerging option for RRMM treatment. The doses were tested from 6.5 to 800 mcg/day [98]. Data from this first-in-human, phase I dose-escalation study (NCT02514239) indicated that at a maximum tolerated dose of 400 mcg/day, the ORR was 70%, including five CR, one VGPR, and one PR. The median time to response was 1 month. Grade ≥ 3 treatment-related AEs included two cases of polyneuropathy and one case of edema. Three out of forty-two patients experienced cytokine release syndrome (CRs) [99]. A dose of 800 mcg/day was considered non-tolerable because of one grade 3 event of CRS and polyneuropathy.

However, AMG 420 has its limitation, i.e., a very short half-life that requires prolonged IV infusion through central venous access. The short half-life, however, can help manage treatment-emergent AEs such as CRS [100]. Further studies are warranted to investigate whether Amgen is beneficial as an early line of therapy. Summary of efficacy and toxicity of BiAbs including AMG 420 in multiple myeloma is given in Table 3.

### 4.4. PF-3135

PF-06863135, also called PF-3135 or elranatamab, is a bispecific humanized mAb (IgG2a), which targets BCMA on MM cells and CD3 on T cells and subsequently causes T-cell-mediated lysis of myeloma cells [101]. Its response has been assessed in patients who have undergone multiple prior MM therapies. Based on the data from the phase I trial (MagnetisMM-1), the results showed promising response rates and a manageable safety profile. This dose-escalation study enrolled 30 patients who received PF-3515 dose of 80–1000 mcg/kg weekly subcutaneously. G ≥3 AEs included lymphopenia (83%), neutropenia (53%), anemia (50%), and thrombocytopenia (37%). The most common treatment-related AE was lymphopenia in 83% of patients followed by CRS (grade 1–2) that occurred in 73% of patients and was dose-dependent. Injection-site reactions occurred in 50% of patients and were less than grade 2. The ORR was 70% at dose of ≥215 µg/kg. VGPR occurred in seven patients, sCR in five patients, and PR/CR in one patient each. [102]. The adverse effects were generally manageable. RP2 dose in this trial was 1000 mcg/k with ORR of 83%. The SC route allowed the administration of higher doses without observing the increased severity of CRS. Pfizer has initiated MagnetisMM-3, a phase II trial to evaluate the efficacy and safety profile of this emerging drug.

### 4.5. REGN5458

Bispecific T-cell engagers (BiTE) is a novel subclass of BiAbs which have the function of binding to T cell and tumor cells, thereby engaging the T cells’ cytotoxic functions in order to induce apoptosis of the cancerous cells [103]. Recently, a new drug, REGN5458, a BCMA × CD3 bispecific antibody, a BiTE, has completed phase I/II trials (NCT03761108) [104]. The study followed the standard 4 + 3 dose-increasing protocol and included all patients that developed RRMM status post >3 systemic therapies. Significant AEs (grades 3–4) occurred in 28.9% of patients, including infection (20%), anemia (8.9%), lymphopenia (6.7%), one patient with acute kidney injury, one patient with syncope, and one patient with transaminitis. ORR was achieved in 35.6% of patients across all doses and 60% at highest dose. VGPR was achieved in 81.3% and CR in 31.3%. Phase II of this trial is currently enrolling and will reveal further details regarding the efficacy of this novel drug.

Of note, a study was recently performed by DiLillo et al. comparing the efficacy of REGN5458 (BiTE) to BCMA-specific CAR-T-cells [105]. The question expressed was warranted since both therapeutics target the same BCMA receptor on the MM cells. In vivo studies showed similar efficacy in their cytotoxic activity against the MM cells. The clear difference between the two was the onset of action. REGN5458 showed a faster onset due to ability to utilize the present T cells for its action versus BCMA-specific CAR-T-cells which require time for activation and transit to the target site.

### 4.6. Talquetamab

GPRC5D (G protein–coupled receptor, class C group 5 member D) is an orphan receptor expressed on malignant plasma cells in MM and is a potential target for chimeric antigen receptor (CAR) T-cell therapy [106]. Talquetamab is an IgG Fc-domain containing BiAbs targeting GPRC5D and CD3 [94]. A phase I trial is currently ongoing by Berdeja et al. [107]. The study has enrolled almost 260 participants with RRMM so far. These patients received talquetamab IV (0.5–180 µg/kg) or SC (5.0–800 µg/kg) once or twice a week. The primary objective of the study is to identify RP2D and determine the AEs at that dose. Data obtained on 8 February 2021 indicated that almost 174 patients had received talquetamab by either IV or SC route. RP2D was identified as 405 µg/kg SC weekly with 10.0 and 60.0 µg/kg step-up doses. The median age of these patients was 61.5 years. The most common grade ≥3 AEs include CRS (4%), neutropenia (54%), anemia (29%), and infections (4%). The response rates at RP2D were as follows: ORR 63% and VGPR 50%. Median time to first response was 1 month and median follow-up time was 6.2 months. The study is currently evaluating the efficacy and safety of RP2D. Total duration of the study is approximately 2 years [108]. Another dose escalation study with talquetamab and teclistamab (Tal + Tec) combination has been ongoing for patients with RRMM. The study is currently in phase I trial and participants will receive Tal + Tec with and without daratumumab to identify the RP2D regimen [109]. In conclusion, these studies are expected to explore the convenient SC and efficacious IV dosing of monotherapy or combination therapy of talquetamab which is an interesting target for RRMM.

**Table 3:** Bispecific antibody summary of efficacy and grade 3 adverse events for treatment of multiple myeloma.

Bispecific Antibody	Study Name/Phase	Single Agent vs. Combination	Efficacy	Most Common Grade ≥ 3 toxicity (%)
			ORR (%)	
Teclistamab	MasjesTEC-1 Phase I [86]	Single Agent	65%	Neutropenia (40%), anemia (27%), thrombocytopenia (18%), fatigue (2%)
CC-93269	Costa et al. Phase I [95]	Single agent	89%	Neutropenia (43%), anemia (37%), infections (30%), thrombocytopenia (17%)
AMG 420	Topp et al. Phase I [98]	Single agent	70%	Cytokine release syndrome (*n* = 3), polyneuropathy (*n* = 2), edema (*n* = 1)
PF-3135/Elranatamab	Levy et al. Phase I [102]	Single agent	33%	Lymphopenia (83%), neutropenia (53%), anemia (50%), thrombocytopenia (37%)
*REGN5458*	Madduri et al. Phase I/II [103]	single agent	35%	Infection (20%), anemia (8.9%), lymphopenia (6.7%), AKI (*n* = 1), syncope (*n* = 1), and transaminitis (*n* = 1)
*Talquetamab*	Berdeja et al. Phase 1 [107]	Single agent	-	CRS (4%), neutropenia (54%), anemia (29%), and infections (4%)

Abbreviations: *n*: number of patients; ORR: overall response rate.

## 5. CAR-T-Cell

### 5.1. Idecabtagene Vicleucel

Idecabtagene vicleucel (ide-cel) is a BCMA-directed CAR-T-cell therapy currently under investigation for its role in RRMM. The KarMMa trial is a phase II trial that evaluated the safety profile and efficacy of ide-cel. As in the other studies, this study included all patients who failed three prior therapies. The regimen included a lymphodepletion phase which included cyclophosphamide and fludarabine for three days; subsequently, ide-cel was given for two days [110]. In this study, grade 3–4 AEs occurred in 99% of patients, including neutropenia (89%), anemia (60%), thrombocytopenia (52%), leukopenia (39%), dumped lymphopenia (27%), and febrile neutropenia (16%). CRS occurred in 5% of patients. Neurotoxic effects occurred in 3% of patients. Of note, the authors do acknowledge that the lymphodepleting chemotherapy given prior to ide-cel may have caused cytopenia. Additionally, AE was not dose-dependent. The patients were divided into three subgroups depending on doses 150, 300, 450 × 10^6^ CAR + T cells. In response to this incremental increase in dose, a CR was observed in 50%, 69%, and 81% of patients, respectively. As of March 2022, the KarMMa trial provided significant evidence to support FDA approval of ide-cel for RRMM [111].

### 5.2. Ciltacabtagene Autoleucel

Ciltacabtagene autoleucel (cilta-cel) is a BCMA-directed CAR T-cell therapy undergoing phase Ib/II studies under the CARTITUDE-1 trial [112]. All the patients went through lymphodepletion with cyclophosphamide and fludarabine prior to receiving cilta-cel. In terms of efficacy, results are as follows: ORR of 97%, sCR of 80.4%, and VGPR of 95%. In terms of significant AEs grade 3–4, neutropenia (85%), anemia (68%), leukopenia (61%), and lymphopenia (50%) were exhibited. The 18-month PFS was 66% and OS 81%, a drop from the 12 months PFS and OS. CARTITUDE-2 is a multi-cohort phase II study, conducted with the intent to determine the effectiveness and safety of cilta-cel in various clinical settings including NDMM, RRMM progressive disease after 1–3 first-line therapies and lenalidomide refractory, RRMM after failure of three therapies, RRMM early relapse after first-line therapy, and s/p ASCT [113]. Currently, results from cohort A are available, patients who had 1–3 prior therapies and were lenalidomide refractory. From cohort A, ORR is 95%, CR is 85%, and VGPR is >95%. These findings are consistent with the CARTITUDE-1 study. Significant grades 3–4 AEs include neutropenia (95%), thrombocytopenia (35%), anemia (45%), lymphopenia (60%), leukopenia (55%), and CRS (10%). Of note, all AEs were manageable as outpatients. Cilta-cel was approved by the FDA on February 2022 for RRMM.

## 6. Conclusions

Understanding the biological mechanism involved in immune evasion of MM cells has led to better disease understanding and immune therapy as a treatment option for MM. The current practice for MM treatment involves immune therapy as a standard approach for RRMM, and it is increasingly being used for NDMM and studies have shown promising results. ADCs consisting of mAbs that carry a cytotoxic payload are directed against various antigens that are very specific for MM cells and represent a targeted chemotherapy approach. ADCs have demonstrated excellent efficacy, and the tumor-directed approaches minimize toxicity to normal tissue. Bispecific Abs is another approach for MM immune therapy that relies on activating the body’s immune system to trigger the killing of neoplastic MM cells. It represents an effective treatment strategy for RRMM patients; in the studies so far, mAbs have also shown favorable results with enhanced efficacy profiles when combined with other anti-MM therapies. These have proven efficacy in patients with both NDMM and RRMM. Immunotherapy can potentially halt disease progression. Immune-directed strategies are being tested to prevent disease progression by treating asymptomatic (but at high risk of progression) forms of SMM. Immunotherapy has revolutionized the treatment paradigm for MM. Questions regarding the safety and tolerability of immunotherapy for MM once thought to be unresolved are eventually finding answers. Immunotherapy continues to be an evolving arena of oncology as myriad mAbs are being investigated in studies in order to seek FDA approval for both NDMM and RRMM.

## Data Availability

Not applicable.
